# Cost-effectiveness of ocriplasmin for the treatment of vitreomacular traction and macular hole

**DOI:** 10.3402/jmahp.v4.31472

**Published:** 2016-06-23

**Authors:** Craig Bennison, Stephanie Stephens, Benedicte Lescrauwaet, Ben Van Hout, Timothy L. Jackson

**Affiliations:** 1 Pharmerit International, York, United Kingdom; 2 Xintera Ltd., St John's Innovation Centre, Cambridge, United Kingdom; 3 School of Health and Related Research, University of Sheffield, Sheffield, United Kingdom; 4 Department of Ophthalmology, School of Medicine, King's College London, London, United Kingdom

**Keywords:** anatomical outcomes, economic evaluation, quality adjusted life year, United Kingdom, vitrectomy, PPV, symptomatic vitreomacular adhesion

## Abstract

**Background:**

If left untreated, vitreomacular traction (VMT) will infrequently improve through spontaneous resolution of vitreomacular adhesion (VMA), and patients remain at risk of further deterioration in vision. The mainstay of treatment for VMT is vitrectomy, an invasive procedure that carries the risk of rare but serious complications and further vision loss. As such, a ‘watch and wait’ approach is often adopted before this surgical intervention is performed. Ocriplasmin (microplasmin) is a potential alternative treatment for patients with symptomatic VMA/VMT that may remove the requirement for vitrectomy.

**Objective:**

The purpose of this study was to evaluate the cost-effectiveness of ocriplasmin for the treatment of VMT in comparison to standard of care.

**Study design:**

A cohort-based computer simulation model was developed, capturing three mutually exclusive subgroups: 1) VMT without epiretinal membrane (ERM) or full thickness macular hole (FTMH), 2) VMT with ERM but no FTMH, and 3) VMT with FTMH. Transition probabilities between health states, utilities, and resource utilisation were estimated based on clinical trial results, the literature, and expert opinion. The cost per quality-adjusted life year (QALY) gained was estimated over a lifetime, using UK unit costs and utilities associated with visual acuity, adverse events, metamorphopsia, and surgical interventions.

**Setting:**

Analyses were conducted from a UK payer perspective.

**Population:**

Transition probabilities for the model were primarily estimated from patient-level data from the combined Phase 3 MIVI-TRUST trials in patients with symptomatic VMA/VMT, including when associated with a FTMH ≤400 µm.

**Intervention:**

Ocriplasmin (microplasmin) is a one-time intravitreal injection designed specifically to release the abnormal traction between the macula and the vitreous and thereby treat VMT, as well as macular hole with persistent vitreous attachment.

**Main outcome measure:**

The main outcome measure of the economic evaluation was cost per QALY.

**Results:**

In all subgroups, ocriplasmin management generated more QALYs: 1) VMT without ERM or FTMH (0.105, (0.036, 0.191)); 2) VMT with ERM but no FTMH (0.041, (0.011, 0.131)); and 3) VMT with FTMH (0.053, (−0.002, 0.113)). The initial treatment costs were partially offset by later savings and net costs were estimated at £1,901 (£1,325, £2,474), £2,491 (£1,067, £2,511), and £1,912 (£1,233, £2,506), respectively. Costs per QALY were estimated at £18,056 (£8,241, £64,874), £61,059 (£8,269, £168,664), and £36,250 (−£144,788, £290,338), respectively. Short-term efficacy parameters were found to be key drivers of results.

**Conclusion:**

Ocriplasmin is most cost-effective in VMT patients without either ERM or FTMH.

Clinically, vitreomacular adhesion (VMA) refers to abnormal, persisting vitreous attachment at or near the fovea, occurring in the context of perifoveal separation (1). If VMA applies sufficient tractional force on the macula, it may distort the macular architecture to cause vitreomacular traction (VMT) or a full-thickness macular hole (FTMH) (2). Both VMT and FTMHs can lead to decreased visual acuity (VA) and metamorphopsia (2). The prevalence of VMT and FTMHs has been estimated at 0.02 and 0.15%, respectively (2).

There are several important gaps in the literature of VMT. Few reports detail the natural history of VMT and there are no large randomised controlled trials of PPV for VMT (3, 4). Traditionally there are two main management options, pars plana vitrectomy (PPV) or observation, the latter for stable mild disease that does not justify the risks of surgery, or in the expectation that in some eyes VMT will resolve spontaneously. Observation may have disadvantages, with a natural history study reporting that only 11% of eyes showed spontaneous resolution over a mean follow-up of 5 years, whereas 64% of eyes lost at least two Snellen lines over this timeframe. VMT can also progress to FTMH during observation. Patients who experience persistent or severe symptomology may undergo PPV. Equally, PPV for VMT may have disadvantages, with only one-third of eyes gaining two or more Snellen lines (3). PPV is associated with postoperative patient burden and the risk of rare but serious complications such as endophthalmitis (5). Postoperative retinal detachment occurs in 2.4% of patients, and 92% of phakic eyes are likely to develop a cataract within 3 years of PPV (6).

Ocriplasmin (microplasmin) is designed specifically to relieve VMA and thereby treat VMT, as well as macular hole (MH) with vitreous attachment. Ocriplasmin enzymatically cleaves collagen, fibronectin, and laminin, leading to vitreous liquefaction and loosening of vitreoretinal attachment (7). Following recent trial publications (ClinicalTrials.gov identifiers NCT00781859 and NCT00798317), an intravitreal injection of ocriplasmin has emerged as an alternative treatment for patients with symptomatic VMA/VMT, including when associated with a FTMH ≤400 µm. The primary outcome of these trials was non-surgical resolution of VMA at Day 28 after a single intravitreal injection of ocriplasmin (7). Importantly, successful anatomic outcomes (VMT resolution and/or FTMH closure) after treatment with ocriplasmin reduced the need for surgical intervention (PPV) (7). Ocriplasmin may thereby be associated with reduced healthcare costs and quality of life (QoL) burdens.

In the present study, the cost-effectiveness of ocriplasmin was determined as a treatment for VMT, based on data from the pivotal ocriplasmin trials (8). Accordingly, we defined three mutually exclusive subgroups: 1) VMT without epiretinal membrane (ERM) or FTMH; 2) VMT with ERM but no FTMH; and 3) VMT with FTMH. The analyses take a UK payer perspective and are aligned with economic evaluation guidance from the National Institute for Health and Care Excellence (NICE) (9).

## Methods

An economic model was constructed to simulate a hypothetical cohort of patients over a lifetime. This model includes estimates of disease progression, as well as the costs and effects associated with two VMT management strategies: 1) a single intravitreal injection of 125 µg ocriplasmin (administered at baseline) and 2) the standard of care (observation and/or PPV as needed). If needed, the ocriplasmin management strategy also allows PPV. This analysis was undertaken after the NICE's Health Technology Appraisal of ocriplasmin (10).

**Fig. 1:**
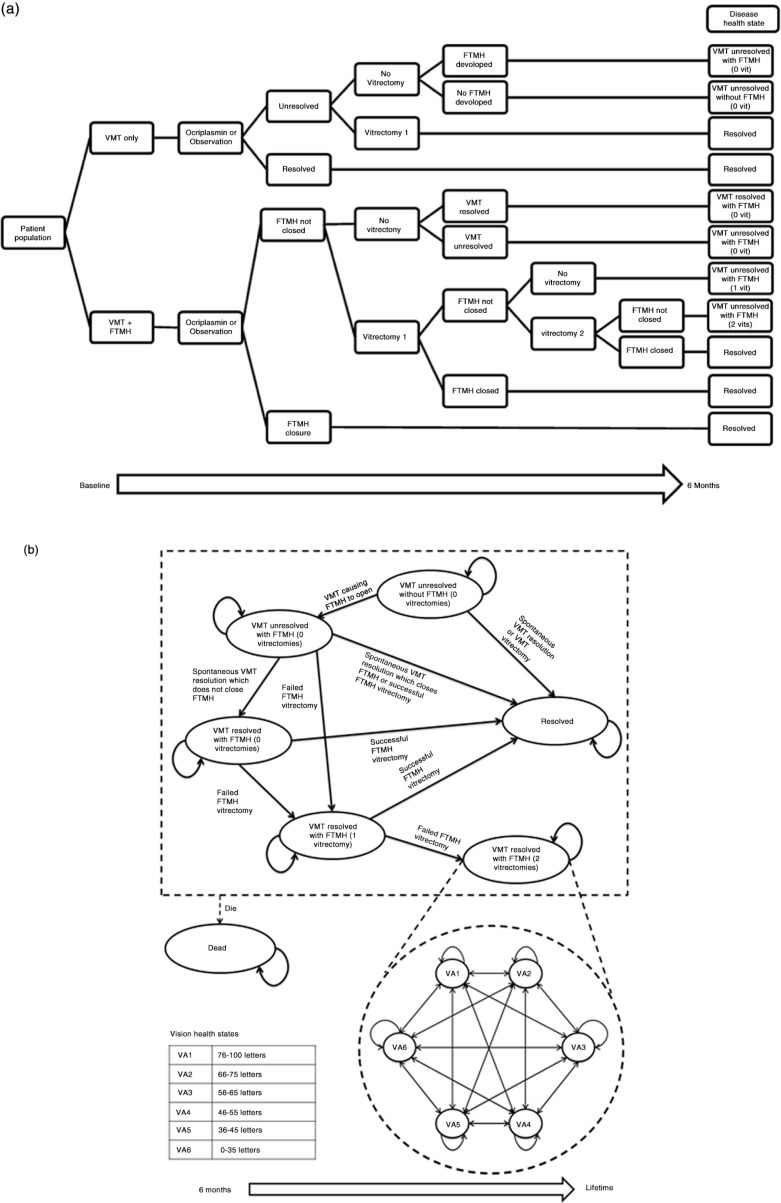
(a) First component of the model: decision-tree structure and resulting disease health states for the start of the extrapolation. (b) Second component of the model: disease health states, vision health states, and associated transitions in the Markov extrapolation. FTMH, full thickness macular hole; VA, visual acuity; VMT, vitreomacular traction.

The model consists of two components. The first component is a short-term decision tree model to simulate participants from the Phase 3 MIVI-TRUST trials (6-month duration), categorised according to whether treatment leads to a successful anatomic outcome (VMT resolution and/or FTMH closure) (Fig. 1a). Patients with VMT alone at baseline have a risk of developing FTMH from persistent VMT. Patients with FTMH at baseline who achieve resolution of VMT may continue to have persistent FTMH. MH closure is the key clinical end point for vision outcomes in patients with FTMH at baseline. In patients without FTMH, VMT resolution is the key clinical end point. The anatomical and PPV components of the short-term model determine the pathway for patients in the longer term.

The second component of the model begins at the end of the decision tree, when patients enter a Markov state–transition extrapolation model (Fig. 1b) to estimate long-term clinical and cost outcomes. This component features two separate health state sets: 1) disease health states to track anatomical status and number of vitrectomies, and 2) vision health states to track patient VA – defined by Early Treatment of Diabetic Retinopathy Study letters read. In total, there are seven disease health states, including death, and six vision health states, most of which cover a mutually exclusive 10-letter range (approximately two Snellen lines, considered clinically significant) (Fig. 1b) (2, 11). For the purpose of this analysis, the worst vision state (VA6, ≤35 letters or ≈6/60) was considered to represent ‘blindness’ (1). Based on the combined status of treatment success, disease progression, and PPV at the end of the initial decision tree, patients are allocated to one disease health state and one vision health state for the start of the extrapolation (Fig. 1a). Short-term event transitions can occur after an event such as successful surgical or pharmacological treatment. Long-term VA health state decline applies to all patients, with patients in the VMT resolved states assumed to have a decline in vision at the same rate as the age-matched general population (12). Patients with unresolved VMT were assumed to experience a faster rate of VA decline to reflect the progressive nature of the disease (13).

Both study-eye and non-study-eye VA were tracked throughout the model. Similarly as applied to patients with resolved VMT, non-study-eye VA declines over time at a rate equivalent to that of VA decline in a general age-matched population (12).

Key assumptions and data sources are listed in detail in the Supplementary file (Supplementary Table 1) and are further described below. Overall, the majority of inputs were obtained through patient-level data analyses from the MIVI-TRUST trial data. This was complemented by a systematic literature search and advice from clinical experts to identify model inputs not obtained from the trial data. Each vision health state was assigned a utility value (14). Evidence suggests that overall vision, and hence QoL, has a stronger relationship with the better-seeing eye (BSE) than the worse-seeing eye (WSE) (15). Approximately 70% of patients from the MIVI-TRUST trials received their injection of ocriplasmin in the WSE. Therefore, a relationship between the WSE and QoL (utility) was modelled. A matrix of utility values, corresponding to each unique BSE–WSE combination, was modelled using study-eye and non-study-eye VA distributions (Supplementary Table 2). Vision health state utility values from the literature were assumed to represent both eyes being in the same VA state (Supplementary Table 3) (14). Utility values for cases where eyes are in different VA states were populated according to the assumption that WSE changes have 30% of the impact that BSE changes have (Supplementary Table 2) (16).

Disutilities were included in the model for adverse events (AEs), metamorphopsia, PPV, and cataract (Supplementary Table 3). Drug and procedure AEs (i.e., ocriplasmin injection or PPV) considered were retinal tear, retinal detachment, elevated intraocular pressure (IOP), vitreous haemorrhage, and cataract. AEs related to the placebo injection were excluded from the analyses.

Based on expert opinion, patients undergoing PPV were considered blind in the study eye for 2 weeks for VMT and 1 month for FTMH. The disutility of metamorphopsia was derived from visual function questionnaire data, which was transformed to utility using a mapping algorithm. Disutilities for retinal detachment, vitreous haemorrhage, and cataract were based on data from the literature. Taking into account the pre-surgical vision loss and cataract recovery, the duration of cataract-related disutility was assumed to be 6 months (Supplementary Table 3).

The transition probabilities describing a patient's anatomical and PPV status, as well as the risk of adverse events, were based primarily on the combined Phase 3 MIVI-TRUST trial data (Table 1, Supplementary Table 3) (7). Trial data were used to estimate the probability of PPV for VMT, the probability of PPV for FTMH, the probability of PPV success for FTMH (defined by anatomical closure of the FTMH), and the probability of FTMH formation from persistent VMT. The probability of requiring a second PPV, and its success rate, were based on clinical expert opinion (17). To increase statistical power, changes in vision due to VMT resolution or FTMH closure were assumed to be equivalent and independent of cause (PPV or non-surgical). The starting age of the simulated cohort was 72 years and 65.8% were female (18). The economic evaluation was performed for three mutually exclusive subgroups, defined by the presence or absence of ERM or FTMH at baseline, as these are distinct diagnoses with different treatment goals (7). Eyes with FTMH are seldom expected to achieve spontaneous FTMH closure; therefore, the observation period for these patients was typically shorter than for eyes with VMT.

**Table 1:** Short-term model inputs and corresponding uncertainty distributions as applied in the sensitivity analyses (5)

I. Short-term model end points (VMT no ERM)			

Input	Deterministic value	95% CI (low)	95% CI (high)
(Ocriplasmin short term) Probability of non-surgical VMT resolution by Day 28	29.79%	23.40%	36.90%
(Ocriplasmin short term) Unresolved VA1 patients at 1 month	29.54%		
(Ocriplasmin short term) Unresolved VA2 patients at 1 month	40.91%		
(Ocriplasmin short term) Unresolved VA3 patients at 1 month	19.70%		
(Ocriplasmin short term) Unresolved VA4 patients at 1 month	6.06%		
(Ocriplasmin short term) Unresolved VA5 patients at 1 month	2.27%		
(Ocriplasmin short term) Unresolved VA6 patients at 1 month	1.52%		
(Ocriplasmin short term) Probability of non-surgical VMT resolution at Month 6, having not had vitrectomy or resolution by Day 28	12.71%	7.30%	20.10%
(Observation short term) Probability of non-surgical VMT resolution by Day 28	7.69%	2.90%	16.00%
(Observation short term) Unresolved VA1 patients at 1 month	34.72%		
(Observation short term) Unresolved VA2 patients at 1 month	40.28%		
(Observation short term) Unresolved VA3 patients at 1 month	13.89%		
(Observation short term) Unresolved VA4 patients at 1 month	8.33%		
(Observation short term) Unresolved VA5 patients at 1 month	1.39%		
(Observation short term) Unresolved VA6 patients at 1 month	1.39%		
(Observation short term) Probability of non-surgical VMT resolution by Month 6, having not had vitrectomy or resolution by Day 28	10.00%	3.80%	20.50%
II. Short-term model end points (VMT with ERM)			

Input	Deterministic value	95% CI (low)	95% CI (high)

(Ocriplasmin short term) Probability of non-surgical VMT resolution by day 28	7.78%	4.20%	12.90%
(Ocriplasmin short term) Unresolved VA1 patients at 1 month	24.68%		
(Ocriplasmin short term) Unresolved VA2 patients at 1 month	40.91%		
(Ocriplasmin short term) Unresolved VA3 patients at 1 month	18.83%		
(Ocriplasmin short term) Unresolved VA4 patients at 1 month	11.69%		
(Ocriplasmin short term) Unresolved VA5 patients at 1 month	1.95%		
(Ocriplasmin short term) Unresolved VA6 patients at 1 month	1.94%		
(Ocriplasmin short term) Probability of non-surgical VMT resolution by Month 6, having not had vitrectomy or resolution by Day 28	7.30%	3.60%	13.00%
(Observation short term) Probability of non-surgical VMT resolution by Day 28	1.61%	0.00%	8.70%
(Observation short term) Unresolved VA1 patients at 1 month	26.23%		
(Observation short term) Unresolved VA2 patients at 1 month	40.98%		
(Observation short term) Unresolved VA3 patients at 1 month	24.59%		
(Observation short term) Unresolved VA4 patients at 1 month	6.56%		
(Observation short term) Unresolved VA5 patients at 1 month	0.00%		
(Observation short term) Unresolved VA6 patients at 1 month	1.64%		
(Observation short term) Probability of non-surgical VMT resolution by Month 6, having not had vitrectomy or resolution by Day 28	4.00%	0.00%	13.70%
III. Short-term model end points (VMT+MH)			

Input	Deterministic value	95% CI (low)	95% CI (high)

(Ocriplasmin short term) Probability of non-surgical MH closure by Day 28	40.57%	31.10%	50.10%
(Ocriplasmin short term) Non-surgical MH closure by Month 6, having not had a vitrectomy or closure by Day 28	17.65%	3.80%	43.40%
(Ocriplasmin short term) Probability of non-surgical VMT resolution by Month 6, having not had MH closure or vitrectomy	50.00%	23.00%	77.00%
(Observation short term) Probability of non-surgical MH closure by Day 28	10.64%	3.50%	23.10%
(Observation short term) Probability of non-surgical MH closure by Month 6, having not had a vitrectomy or closure by Day 28	25.00%	7.30%	52.40%
(Observation short term) Probability of non-surgical VMT resolution by Month 6, having not had MH closure or vitrectomy	58.33%	27.70%	84.80%

CI, confidence interval; ERM, epiretinal membrane; MH, macular hole; VA, visual acuity; VMT, vitreomacular traction.

Costs associated with adverse events consider a combination of Healthcare Resource Group codes (Supplementary Table 3). The cost of raised IOP considers only treatment costs, which were sourced from the British National Formulary. The cost of blindness was calculated as the sum of low-vision rehabilitation, depression, and hip fracture/replacement costs (19). The cost of PPV includes the surgical procedure, follow-up visits, and the recovery burden. A recent UK study estimated that 40.5% of PPVs were combined with cataract surgery (6). Hence, the cost and QoL impact of cataract surgery were weighted as such. Unit costs represent 2012 values (Supplementary Table 3). Both costs and quality-adjusted life years (QALYs) were discounted at 3.5% per annum following NICE guidelines.

In order to compare the strategies, a cohort was simulated through the model assuming treatment with ocriplasmin and then repeated assuming standard of care management, followed by PPV as needed. Extrapolations were based on the anatomical distribution of patients at 6 months (Fig. 1a), which was driven by treatment. The main outcome measure of the model is the incremental cost-effectiveness ratio (ICER), expressed as the additional cost per QALY gained, capturing both the costs and benefits to the patient.

**Fig. 2:**
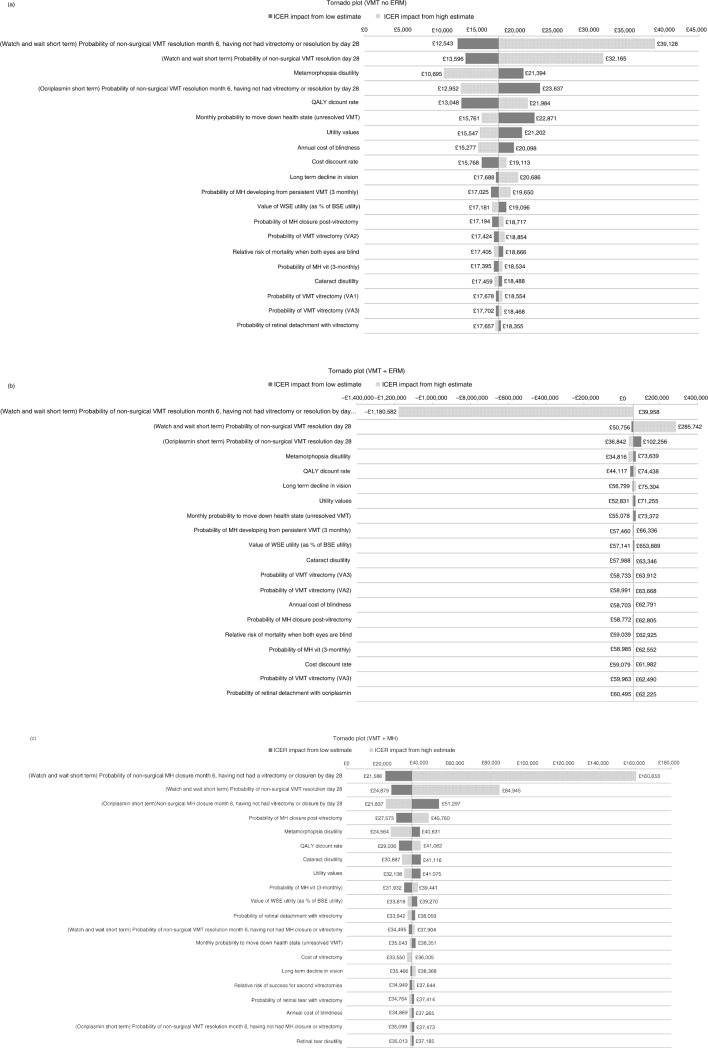
One-way sensitivity analyses presented as tornado plots for each subgroup: (a) VMT without ERM or FTMH (VMT no ERM); (b) VMT with ERM (VMT+ERM); (c) VMT with FTMH (VMT+FTMH). CE, cost-effectiveness; ERM, epiretinal membrane; FTMH, full-thickness macular hole; ICER, incremental cost-effectiveness ratio; VMA, vitreomacular adhesion; VMT, vitreomacular traction.

**Fig. 3:**
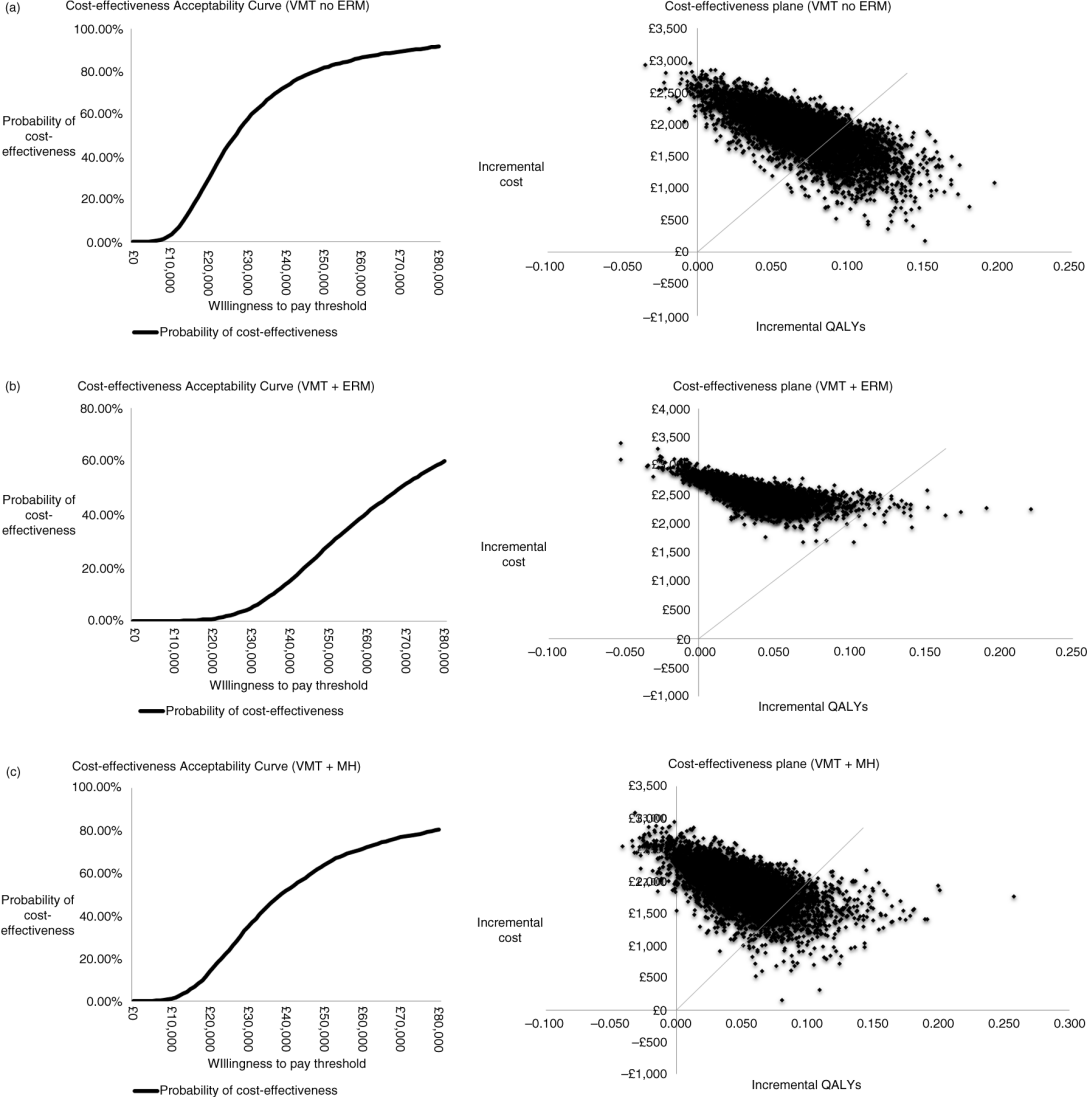
Cost-effectiveness acceptability curves and scatterplots for each subgroup: (a) VMT without ERM or FTMH (VMT no ERM), (b) VMT with ERM (VMT+ERM), (c) VMT with FTMH (VMT+FTMH). ERM, epiretinal membrane; FTMH, full thickness macular hole; VMT, vitreomacular traction.

Scenario analyses were conducted to examine the effect of several assumptions (Supplementary Table 4). The impact of key assumptions on the ICER were tested for the following: 1) maximum time limit for PPVs to occur into extrapolation phase; 2) immediate PPV for patients with FTMH; 3) patients with FTMH experience a visual decline at the same rate as patients with VMT; 4) alternate published source of utility values; 5) adjustment of the utility impact of changes in the WSE relative to changes in the BSE; 6) AE rates for ocriplasmin and PPV; 7) increase of metamorphopsia disutility; and 8) model time horizon. In addition, distributions were fitted around each parameter (Supplementary Table 3) and one-way sensitivity analyses and probabilistic sensitivity analyses were conducted to assess the uncertainty in the model (Figs. 2a–c and 3a–c). To test validity, health state distributions at the end of the trial were compared to the modelled health state distributions at 6 months (7).

## Results

Ocriplasmin was associated with increased QoL. Table 2 presents the discounted costs and QALYs per subgroup. The cost of the ocriplasmin injection was partially offset by savings from avoided vitrectomies and reduced incidence of cataracts, adverse events, and blindness. Based on the assumption that PPV could follow either management strategy if the underlying condition(s) did not resolve, the ocriplasmin-to-standard-of-care ICERs were £18,056/QALY (£8,241, £64,874), £61,059/QALY (£8,269, £168,664), and £36,250/QALY (−£144,788, £290,338), for the VMT without ERM or FTMH, VMT with ERM, and VMT with FTMH subgroups, respectively. All costs, QALYs, and subsequent ICERs were expressed per patient over a lifetime period.

**Table 2:** Summary of discounted costs and quality-adjusted life year breakdown in patients in each subgroup who received standard of care or ocriplasmin

		VMT no ERM			VMT with ERM			VMT with FTMH		
				
		Standard of care	Ocriplasmin	Incremental	Standard of care	Ocriplasmin	Incremental	Standard of care	Ocriplasmin	Incremental
Costs	Drug and administration costs	£0	£2,617	£2,617	£0	£2,617	£2,617	£0	£2,617	£2,617
	Vitrectomy and cataract costs	£977	£725	−£252	£1,190	£1,085	−£105	£1,754	£1,197	−£557
	Adverse event costs	£188	£154	−£34	£212	£208	−£4	£329	£239	−£90
	Monitoring costs	£1,040	£1,023	−£17	£1,158	£1,277	£119	£656	£701	£45
	Blindness costs	£1,983	£1,570	−£413	£1,703	£1,567	−£136	£614	£511	−£103
	Total	£4,188 (£2,705, £8,928)	£6,088 (£5,273, £11,317)	£1,901 (£1,325, £2,474)	£4,263 (£2,455, £8,891)	£6,755 (£4,688, £10,441)	£2,491 (£1,067, £2,511)	£3,353 (£2,187, £6,863)	£5,266 (£4,360, £8,620)	£1,912 (£1,233, £2,506)
QALYs	Visual acuity state QALYs	7.047	7.130	0.083	7.148	7.179	0.031	7.424	7.456	0.032
	Vitrectomy and cataract disutility^a^	−0.016	−0.012	0.004	−0.028	−0.025	0.002	−0.035	−0.024	0.011
	Adverse event disutility^a^	−0.009	−0.007	0.002	−0.010	−0.009	0.000	−0.015	−0.011	0.004
	Metamorphopsia disutility^a^	−0.066	−0.049	0.016	−0.074	−0.067	0.007	−0.024	−0.018	0.006
	Total	6.956 (5.953, 8.168)	7.062 (5.985, 8.208)	0.105 (0.036, 0.191)	7.036 (5.845, 8.056)	7.077 (5.934, 8.138)	0.041 (0.011, 0.131)	7.351 (6.242, 8.472)	7.403 (6.284, 8.512)	0.053 (−0.002, 0.113)

Discrepancies in incremental results are due to rounding.

a Less disutility with ocriplasmin treatment compared to standard of care equals positive incremental QALY. ERM, epiretinal membrane; FTMH, full thickness macular hole; QALY, quality-adjusted life year; VA, visual acuity; VMT, vitreomacular traction.

Short-term efficacy parameters (non-surgical VMT resolution or FTMH closure) were key drivers of cost-effectiveness results because these determined the patient's disease health state at the start of extrapolation and, hence, the course of disease progression throughout the extrapolation period. Assessing cost-effectiveness according to three different subgroups resulted in small sample sizes, which were reflected in the wide confidence intervals for the treatment efficacy parameters. In Fig. 2b, the extreme ICER impact when applying the high estimate (top row) is due to that scenario producing negative incremental QALYs. In all subgroups, the majority of iterations from the probabilistic analyses demonstrated increased QALYs with ocriplasmin, but for additional costs (Fig. 3a–c).

Six-monthly health state distributions were comparable for each subgroup when comparing the model outcomes with the trial results, supporting the internal validity of the model (7).

## Discussion and conclusion

This study aimed to estimate the clinical and cost-effectiveness of ocriplasmin for the treatment of three clinically distinct patient subgroups, namely VMT without ERM, VMT with ERM, and VMT with FTMH. These subgroups were defined by baseline ocular prognostic factors (the presence or absence of ERM, or of MH), treatment goals (VMT resolution *versus* MH closure), different efficacy profiles of ocriplasmin, and the expected treatment pathway (ocriplasmin use as an alternative to surgery, or for ‘watch and wait’ patients who have severe symptoms but are not eligible for surgery *versus* ocriplasmin used during the wait for surgery, without delaying the surgery).

Ocriplasmin was compared to standard of care in a UK-based model that used a lifetime horizon to capture long-term vision changes associated with treatment. The model incorporated the likelihood of PPV occurring if the underlying condition(s) did not resolve or symptoms remained. Ocriplasmin treatment was associated with higher QALY gains and incremental costs in all three subgroups when compared to standard of care. Based on commonly accepted UK cost-effectiveness willingness-to-pay thresholds of £20,000 to £30,000 per QALY, the results seen in the VMT without either ERM or FTMH subgroup were considered highly cost-effective from a UK perspective (9).

Savings from the avoidance of blindness partially offset the cost of ocriplasmin in this subgroup. The majority of QALYs gained are from the lifetime accrual of VA benefits. Savings from avoidance of vitrectomies and cataract operations partially offset the cost of ocriplasmin in the VMT with FTMH subgroup. The QALY gains in this subgroup were primarily driven by the lower incidence of surgical interventions. The lack of cost-effectiveness in patients with ERM is primarily due to low rates of efficacy in this subgroup (7).

The scenario analyses suggest that the results from this cost-effectiveness analysis were reasonably robust to changes in some of the model's assumptions.

Sensitivity analyses demonstrated that inputs pertaining to short-term clinical outcomes, such as VMT resolution, FTMH closure, and need for PPV, were key model drivers across all subgroups. This confirms the validity of modelling cost-effectiveness by clinically relevant subgroups based on the presence of ERM or FTMH. Further investigation of the impact of using alternative parameters showed that the largest areas of uncertainty in the VMT without ERM subgroup were related to the impact of metamorphopsia disutility, VA decline in unresolved VMT patients, VA-associated utility, and cost of blindness, with higher estimates reducing the ICER. In the VMT plus MH subgroup, reduction in the likelihood of further surgical procedures (MH closure post-PPV) had the largest increase on the ICER. Similarly, lower estimates for disutility of metamorphopsia or cataract and for utility of VA health states increased the ICER.

The MIVI-TRUST trials were designed in line with FDA requirements. The comparator arm was based on a placebo injection and/or PPV as needed. This was likely to bias against ocriplasmin, as an invasive treatment is not part of the current standard of care. The efficacy results seen in the placebo-injection arm are likely to be higher than those seen in clinical practice. Therefore, any potential bias from having to adopt the placebo injection rather than true observation is working against ocriplasmin due to a reduced efficacy gap.

The ERM status of the patient was assumed not to affect the treatment goals or pathways, which may have increased the uncertainty around cost-effectiveness estimates in this patient population. Excluding ERM from the modelled treatment pathways may have favoured ocriplasmin. This is because the model does not account for the possibility of ERM in otherwise resolved patients (i.e., those without VMT or MH). Patients with ERM are less likely to experience visual decline similar to that of the general population (as assumed for resolved patients), and they are likely to experience symptoms such as metamorphopsia. In addition, the decision to proceed with surgery may be affected by the symptoms associated with ERM and its impact on daily living, as well as by the duration of ERM. On the other hand, the impact of ERM is indirectly captured within the covariates for visual acuity at baseline and 6 months, and the alternative option would have been to increase the complexity of the model as well as the number of assumptions required.

To our knowledge, the present study is the first health-economic evaluation comparing ocriplasmin with standard of care in VMT patients using a state-transition model, where the decision problem was conceptualised in terms of disease and vision health states and transitions among these states, and in terms of other characteristics relevant to the decision problem, including metamorphopsia. State-transition models are appropriate for decision problems that require a lifetime horizon and are one of the most widespread modelling techniques in clinical decision analysis and health economic evaluation (20).

Compared to previous ophthalmology models, our model also tracks patient vision over time with a set of discrete, mutually exclusive health states defined by VA (21–23). A major strength of our study is that we explicitly model VA of both the BSE and WSE, allowing appropriate assignment of utilities. This unique approach was designed to acknowledge evidence that the BSE has a stronger relationship with vision-related QoL than the WSE, whilst simultaneously taking into consideration that patients injected in their WSE would still derive some benefit from the treatment (especially in the case where a WSE becomes a BSE after treatment). Given the clinical relevance of metamorphopsia as a symptom in patients with FTMH and symptomatic VMA/VMT, we also attempted to model the impact of anatomical outcomes on metamorphopsia and the impact of metamorphopsia on QoL (9, 24).

This modelling study followed recommended methodological practices and standards in health economics (25). Unlike the recent cost evaluation for treatment of VMA and MHs (26), the first step in our study was to conduct a systematic review of the literature, which was needed to populate the model parameters related to efficacy end points for the comparative treatment options. A systematic literature review is important to ensure both treatment options are interchangeable in the treatment pathway and are used in a population with similar baseline characteristics, such as baseline level of disease stage and visual acuity (27). Currently, the single robust source for randomised prospective evidence is the MIVI-TRUST clinical trials, which was the primary source of evidence used in this study.

In conclusion, ocriplasmin therapy produces health-related QoL benefits when compared with the standard of care. The results suggest that the cost-effectiveness of ocriplasmin is highest for VMT without ERM patients. Following NICE technology appraisal methods and requirements, the cost-effectiveness estimates predicted by the current model in patients with VMT with ERM or Stage II MHs up to 400 microns with persisting VMA are less certain (as demonstrated in Figure 2), and further research may be warranted. Cost per QALY thresholds are country-specific and this model can be adapted for assessing cost-effectiveness in other countries. If thresholds as calculated by the WHO project Choosing Interventions That Are Cost-Effective are assumed, the use of ocriplasmin in patients with VMT alone or with VMT and FTMH may be considered to be cost-effective (28).

## Supplementary Material

Cost-effectiveness of ocriplasmin for the treatment of vitreomacular traction and macular hole
