# Supplementary material for: Cost-effectiveness of ocriplasmin for the treatment of vitreomacular traction and macular hole
Source: J Mark Access Health Policy. 2016 Jun 23;4:10.3402/jmahp.v4.31472. doi: 10.3402/jmahp.v4.31472 (PMC4920942; doi:10.3402/jmahp.v4.31472)
Supplement: Cost-effectiveness of ocriplasmin for the treatment of vitreomacular traction and macular hole [file JMAHP-4-31472-s001.docx]

# Supplementary Material

#### The supplementary material includes tables outlining details of the assumptions, global vision utilities applied in the model, uncertainty distributions around non-subgroup specific model parameters, results of scenario analyses, and a figure that provides a visual representation of the patient subgroups analysed.

##### Supplement Table 1. Key assumptions and data sources

| **Input** | **Assumption** | **Data source** |
| --- | --- | --- |
| Events | Patients undergo a maximum of 2 PPVs. | A UK study indicates 0.2% of patients undergo 3 or more PPVs for FTMH (1). |
|  | PPV successfully resolves VMT in 100% of cases. | Clinical expert opinion that VMT surgery seldom fails to resolve VMT. |
|  | PPV occurs within 2 years into the extrapolation phase. | Clinical expert opinion that if patients have not resolved (either surgically or non-surgically) after 2 years they will not undergo further treatment. |
|  | Spontaneous resolution occurs within 6 months. | MIVI-TRUST data.  Patients had a mean disease duration of ~9 months at study baseline and are therefore unlikely to experience resolution beyond what was observed in the study. |
|  | Probability of AEs associated with ocriplasmin and/or PPV, including retinal detachment, retinal tear, vitreous haemorrhage, and increased intraocular pressure. | MIVI-TRUST data. |
|  | Probability of cataract extraction in pseudophakic patients, post vitrectomy | The probability of developing a cataract after vitrectomy was estimated from a UK study that estimated a 3-year cataract probability of 92%. (1) The proportions of pseudophakic patients by subgroup were estimated from MIVI-TRUST data. |
|  | Patient mortality | UK National Life Tables. (2) |
|  | Mortality HR of 1.54 for patients whose BSE was in VA6 (blindness) | Data from Christ et al, 2008. (3) |
| Patient management and follow-up | Patients with a PPV have 4 follow-up visits, with an OCT performed at each visit. | Advice from clinical experts. |
|  | Patients with ocriplasmin have 1 visit for injection plus 1 post-injection follow-up visit, including 1 OCT scan. |  |
|  | Patients with unresolved condition are monitored with 1 visit every 3 months, including 1 OCT scan per visit, for up to 2 years into the extrapolation. |  |
|  | OCT assessment is required for VMT diagnosis in all patients |  |
| Adverse events | AEs associated with ocriplasmin and/or PPV included retinal detachment, retinal tear, vitreous haemorrhage, and increased intraocular pressure. | Clinical expert opinion. AE rates were based on MIVI-TRUST data. |
|  | A proportion of patients with persistent VMT or FTMH experience on-going metamorphopsia. | As observed in OASIS trials. Data on file. |
|  |  |  |
| Long-term vision | NSE VA declined over time at a rate equivalent to VA decline in a general age-matched population (and as applied to patients with resolved VMT). | Assumption. Validated by clinical expert opinion. |
|  | Using SE and NSE vision health state distributions permit calculation of vision health state distributions for both the BSE and WSE. | Novel approach to avoid the simplifying assumption of previous Markov models that the model cohort has either a BSE or WSE treated, the fellow eye, effectively, being ignored. |
|  | Patients with resolved VMT but persistent FTMH experience long-term VA decline at a rate similar to a resolved patient | The VA of patients with resolved sVMT was assumed to decline at the same rate as the age-matched general population. (4) Clinical experts have supported this assumption. In the absence of published evidence and confirmation based on expert opinion that progression of VA is worse for patients with MH compared with sVMT alone, long-term VA decline was conservatively assumed to be the same for all patients with resolved sVMT, regardless of MH status. |
|  | Long-term VA decline associated with VMT | Rate of VA decline over time was estimated from the literature. (5) |
|  | Long-term VA decline in resolved population follows VA decline in a general age-matched population | Study with vision data from a general Finnish population. (4) |
| VMT / FTMH resolution | Short-term, one-cycle vision health state transitions associated with VMT resolution or FTMH closure | MIVI-TRUST data.  Estimated by comparing baseline VA between patients who had FTMH at baseline versus those who did not, assuming that any difference in the vision distribution was a result of FTMH status.  Validated by clinical experts. |
|  | Short-term vision health state transitions associated with FTMH formation |  |
|  | Changes in vision due to VMT resolution or FTMH closure are equivalent and independent of cause (PPV or non-surgical). |  |
| Utilities | Each VA health state is assigned a utility value | Czoski-Murray et al, 2009. In which specially designed lenses were used to simulate vision impairment in a group of adults. (6) |
| Disutilities | Patients with PPV are considered blind in their study eye during the 2 weeks following surgery; disutility associated with this ranges from 0.03 to 0.07, depending on vision before blindness. | Clinical expert opinion |
|  | Disutilities:  Retinal detachment 0.13;  Vitreous haemorrhage 0.02 | Busbee et al, 2002. (7)  Brandle et al, 2007. (8) |
|  | Disutility of metamorphopsia was estimated to be 0.017. | Derived from visual function questionnaire data by Fukuda et al, 2009 and transformed to a utility value using a mapping algorithm from Payakachat et al, 2009. (9,10) |
|  | Disutility for cataract surgery of 0.14 for 3 months. Duration of disutility extended to 6 months to account for the disutility associated with pre-surgical vision loss from cataract. | Disutility estimated from Gupta et al, 2007. (11)  Duration of disutility based on clinical expert opinion. |
| Costs | Costs associated with AEs take into account a combination of HRG codes. | Published UK NHS reference costs 2011/2012. (12) |
|  | Cost of raised IOP includes only treatment costs. | British National Formulary, September 2012. (13) |
|  | Cost of blindness combines various cost components incl. low-vision rehabilitation, depression, and hip fracture/replacement. | Meads and Hyde, 2003. (14) |
|  | Cost of cataract surgery takes into account that 40.5% of PPVs are combined with cataract surgery. | UK study that found around 40.5% of vitrectomies are performed as combined vitrectomy plus cataract surgeries. Jackson et al, 2013. (1) |

OCT: optical coherence tomography; SE: study eye; NSE: non–study eye; BSE: better-seeing eye; WSE: worse-seeing eye; HR: hazard rate; HRG: Healthcare Resource Group.

##### Supplement Table 2. Global Vision Utility Matrix.

| **Utility** | | **BSE** | | | | | |
| --- | --- | --- | --- | --- | --- | --- | --- |
|  |  |  |  |  |  |  |  |
|  |  | **VA1** | **VA2** | **VA3** | **VA4** | **VA5** | **VA6** |
| **WSE** | **VA1** | 0.7900 |  |  |  |  |  |
|  | **VA2** | 0.7623 | 0.6700 |  |  |  |  |
|  | **VA3** | 0.7438 | 0.6515 | 0.5900 |  |  |  |
|  | **VA4** | 0.7277 | 0.6354 | 0.5738 | 0.5200 |  |  |
|  | **VA5** | 0.7092 | 0.6169 | 0.5554 | 0.5015 | 0.4400 |  |
|  | **VA6** | 0.7000 | 0.6077 | 0.5462 | 0.4923 | 0.4308 | 0.4000 |

Abbreviations: BSE, better-seeing eye; VA, visual acuity; WSE, worse-seeing eye.

The vision health state utility values provided by Czoski-Murray were assumed representative of “global vision,” where both eyes are in the same VA state. (6) Global vision and associated utility values are shown on the lead diagonal of the utility matrix in Table 1 (grey-shaded diagonal). The remaining cells were populated according to the assumption that WSE changes have 30% of the impact that BSE changes have, as recommended by the National Institute of Health and Care Excellence (NICE) review of fluocinolone acetonide. (15)

Supplement Table 3. Non-subgroup specific model inputs and corresponding uncertainty distributions as applied in the sensitivity analyses

| **Input type** | **Input** |  | | | |
| --- | --- | --- | --- | --- | --- |
|  |  | **Deterministic value** | **95% CI (low)** | **95% CI (high)** | **Source** |
|  | % Female | 65.80% | 56.22% | 74.77% | MIVI-TRUST data |
| **Long-term vision** | Probability to decline a health state (VMT resolved) | 2.04% | 0.25% | 5.58% | Assumptions based on (4) |
|  | Probability to decline a health state (VMT unresolved) | 12.92% | 7.09% | 20.16% | Assumptions based on (5) |
| **Costs** | Cost of vitrectomy | £1 770.14 | £849 | £3 024 | (12) |
|  | Cost of ocriplasmin administration | £117.00 | £56 | £200 | (12) |
|  | Cost of cataract | £506.35 | £243 | £865 | (12) |
|  | Cost of blindness (annual) | £6 496.00 | £3 115 | £11 098 | (14) |
|  | Cost of OCT | £54.29 | £26 | £93 | (16) |
|  | Cost of follow-up visit | £80.00 | £38 | £137 | (12) |
|  | Cost of retinal tear | £423.55 | £203 | £724 | (12) |
|  | Cost of retinal detachment | £2 012.00 | £965 | £3 437 | (12) |
|  | Cost of elevated intraocular pressure | £40.65 | £19 | £69 | Assumptions based on (11) |
|  | Cost of vitreous haemorrhage | £1 852.00 | £888 | £3 164 | (12) |
| **Utilities** | Value of WSE utility (as % of BSE utility) | 0.300 | 0.190 | 0.424 | Assumption |
|  | Vitrectomy surgery disutility duration (VMT) (months) | 0.500 | 0.407 | 0.603 | Expert opinion |
|  | Vitrectomy surgery disutility duration (FTMH) (months) | 1.000 | 0.814 | 1.205 | Expert opinion |
|  | Cataract disutility per 3 months | 0.056 | 0.020 | 0.109 | (11) |
|  | Retinal detachment disutility per 3 months | 0.068 | 0.027 | 0.124 | (7) |
|  | Vitreous haemorrhage disutility per 3 months | 0.002 | 0.000 | 0.014 | (8) |
|  | Metamorphopsia disutility (3 monthly) | 0.004 | 0.000 | 0.023 | Assumptions based on (9) |
| **Event rates** | Probability of cataract extraction in pseudophakic patients, post vitrectomy | 92.00% | 85.94% | 96.46% | (1) |
|  | Probability of retinal tear with vitrectomy | 13.23% | 5.37% | 23.92% | MIVI-TRUST data |
|  | Probability of retinal detachment with vitrectomy | 13.23% | 5.37% | 23.92% | MIVI-TRUST data |
|  | Probability of elevated intraocular pressure with vitrectomy | 26.46% | 15.22% | 39.52% | MIVI-TRUST data |
|  | Probability of vitreous haemorrhage with vitrectomy | 3.31% | 0.29% | 9.76% | MIVI-TRUST data |
|  | Probability of retinal tear with ocriplasmin | 0.22% | 0.01% | 0.80% | MIVI-TRUST data |
|  | Probability of retinal detachment with ocriplasmin | 0.43% | 0.05% | 1.20% | MIVI-TRUST data |
|  | Probability of elevated intraocular pressure with ocriplasmin | 2.37% | 1.19% | 3.94% | MIVI-TRUST data |
|  | Probability of vitreous haemorrhage with ocriplasmin | 0.22% | 0.01% | 0.80% | MIVI-TRUST data |
|  | Probability of VMT PPV (VA1) | 2.28% | 1.16% | 3.76% | MIVI-TRUST data |
|  | Probability of VMT PPV (VA2) | 3.26% | 1.89% | 4.99% | MIVI-TRUST data |
|  | Probability of VMT PPV (VA3) | 4.63% | 2.97% | 6.64% | MIVI-TRUST data |
|  | Probability of VMT PPV (VA4) | 6.51% | 4.52% | 8.83% | MIVI-TRUST data |
|  | Probability of VMT PPV (VA5) | 9.06% | 6.70% | 11.73% | MIVI-TRUST data |
|  | Probability of VMT PPV (VA6) | 12.40% | 9.66% | 15.43% | MIVI-TRUST data |
|  | Probability of MH PPV (3-monthly) | 43.10% | 33.77% | 52.68% | MIVI-TRUST data |
|  | Relative risk of success for second vitrectomies | 50.00% | 40.22% | 59.78% | Expert opinion |
|  | Probability of MH closure post vitrectomy | 87.20% | 78.69% | 93.78% | MIVI-TRUST data |
|  | Probability of undergoing a second vitrectomy | 54.50% | 44.68% | 64.15% | Expert opinion |
|  | Probability of MH developing from persistent VMT (3-monthly) | 6.92% | 3.74% | 10.97% | MIVI-TRUST data |
|  | Metamorphopsia prevalence in MH patients | 88.50% | 81.01% | 94.30% | Data on file. 2013. |
|  | Metamorphopsia prevalence in VMT patients | 69.70% | 61.26% | 77.53% | Data on file. 2013. |
|  | Relative risk of mortality when both eyes are blind | 1.54 | 1.26 | 1.84 | (3) |

Abbreviations: BSE, better-seeing eye; CI, confidence interval; FTMH, full-thickness macular hole; MH, macular hole; OCT, optical coherence tomography; PPV, pars plana vitrectomy; QALY, quality-adjusted life year; VMT, vitreomacular traction; WSE, worse-seeing eye.

##### Supplement Table 4. Scenario Analyses

|  | **VMT No ERM ICER** | **VMT+ERM ICER** | **VMT+FTMH ICER** |
| --- | --- | --- | --- |
| Base case | £18 056 | £61 059 | £36 250 |
| Adjustment of time limit of vitrectomies |  |  |  |
| 1 year | £14 173 | £47 108 | £36 250 |
| 5 years | £27 338 | £93 057 | £40 932 |
| FTMH patients proceed straight to vitrectomy |  |  | £42 512 |
| Brown 1999 (1) used as source for utility values | £22 902 | £77 620 | £43 970 |
| Adjustment in the utility impact of changes in the WSE relative to changes in the BSE |  |  |  |
| 0% of BSE utility impact | £21 910 | £80 019 | £48 239 |
| 10% of BSE utility impact | £20 211 | £71 298 | £42 679 |
| 20% of BSE utility impact | £18 984 | £65 361 | £38 939 |
| 40% of BSE utility impact | £17 330 | £57 799 | £34 225 |
| 50% of BSE utility impact | £16 747 | £55 242 | £32 645 |
| 60% of BSE utility impact | £16 268 | £53 184 | £31 377 |
| 70% of BSE utility impact | £15 867 | £51 491 | £30 337 |
| 80% of BSE utility impact | £15 527 | £50 074 | £29 469 |
| 90% of BSE utility impact | £15 235 | £48 871 | £28 734 |
| 100% of BSE utility impact | £14 982 | £47 836 | £28 102 |
| Adverse event rates adjusted for both ocriplasmin and vitrectomy |  |  |  |
| +25% | £17 901 | £60 883 | £35 109 |
| -25% | £18 213 | £61 237 | £37 439 |
| Increase of metamorphopsia disutility to 0.14 (2) | £8 481 | £27 303 | £20 362 |
| Adjustments in time horizon |  |  |  |
| 5 years | £65 466 | £197 319 | £67 289 |
| 10 years | £34 147 | £104 810 | £49 750 |
| 15 years | £23 724 | £75 978 | £41 489 |
| 20 years | £19 833 | £65 703 | £37 977 |

Abbreviations: BSE, better-seeing eye; ICER, incremental cost-effectiveness ratio; FTMH, full thickness macular hole; VA, visual acuity; VMT No ERM, vitreomacular traction patients with no epiretinal membrane; VMT+ERM, vitreomacular traction patients with epiretinal membrane; VMT+FTMH, vitreomacular traction patients with full thickness macular hole; WSE, worse-seeing eye.

**
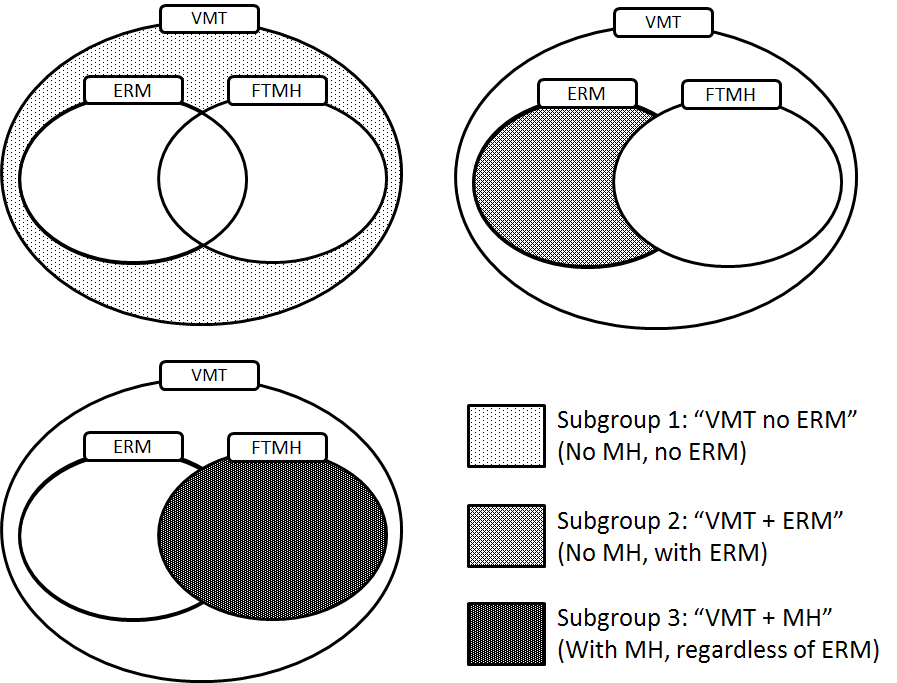
**

**Supplement Figure 1.** Patient subgroups featured in the economic evaluation. While VMT and FTMH may overlap at the same time in the same eye, they are distinct diagnoses with different treatment goals (VMT resolution or FTMH closure, respectively). Ocriplasmin has a different efficacy profile in each subgroup, particularly relevant to the ERM subgroup.

Abbreviations: CE, cost-effectiveness; ERM, epiretinal membrane; FTMH, full-thickness macular hole; VMA, vitreomacular adhesion; VMT, vitreomacular traction.

# Supplementary Material References

(1) Jackson TL, Donachie PH, Sparrow JM, Johnston RL. United Kingdom national ophthalmology database study of vitreomacular surgery: report 2, macular hole. *Ophthalmology*. 2013;**120(3)**:629-634.

(2) Office of National Statistics. Interim Life Tables, 2008-2010. [Internet]. [cited 2013 January 22]. Available from: <http://www.ons.gov.uk/ons/rel/lifetables/interim-life-tables/2008-2010/sum-ilt-2008-10.html>.

(3) Christ S, Lee D, Lam B, Zheng D, Arheart K. Assessment of the Effect of Visual Impairment on Mortality through Multiple Health Pathways: Structural equation modeling. *Investigative Ophthalmology & Visual Sciences*. 2008;**49(8)**:3318-3323

(4) Laitinen A, Koskinen S, Harkanen T, Reunanen A, Laatikainen L, Aromaa A. A nationwide population-based survey on visual acuity, near vision, and self-reported visual function in the adult population in Finland. *Ophthalmology*. 2005;**112(12)**:2227-2237.

(5) Hikichi T, Yoshida A, Trempe CL. Course of Vitreomacular Traction Syndrome. *Am J Ophthalmol*. 1995;**119**:55-61.

(6) Czoski-Murray C, Carlton J, Brazier J, Young T, Papo NL, Kang HK. Valuing condition-specific health states using simulation contact lenses. *Value Health.* 2009;**12(5)**:793-799.

(7) Busbee B, Brown M, Brown G, Sharma S. Incremental cost-effectiveness of initial cataract surgery. *Ophthalmology.* 2002:**109(3)**:606-613.

(8) Brändle M, Azoulay M, Greiner RA. Cost-effectiveness and cost-utility of insulin glargine compared with NPH insulin based on a 10-year simulation of long-term complications with the Diabetes Mellitus Model in patients with type 2 diabetes in Switzerland. *International journal of clinical pharmacology and therapeutics*. 2007:**45(4)**:203-220.

(9) Fukuda S, Okamoto F, Yuasa M, Kunikata T, Okamoto Y, Hiraoka T, Oshika T. Vision-related quality of life and visual function in patients undergoing vitrectomy, gas tamponade and cataract surgery for macular hole. *Br J Ophthalmology*. 2009;**93(12**):1595-1599.

(10) Payakachat N, Summers K, Pleil A, Murawski M, Thomas J, Jennings K, Anderson J. Predicting EQ-5D utility scores from the 25-item National Eye Institute Vision Function Questionnaire (NEI-VFQ 25) in patients with age-related macular degeneration. *Quality of Life Research*. 2009 801-813.

(11) Gupta O, Brown G, Brown M. A value-based medicine cost-utility analysis of idiopathic epiretinal membrane surgery. *Am J Ophthalmology*. 2007;**145(5**):923-928

(12) NHS. National Schedule of Reference Costs 2011/2012

(13) British National Formulary, September 2012. Available from https://www.bnf.org/

(14) Meads C and Hyde C. What is the cost of blindness? *Br J Ophthalmology*. 2003;**87(10):**1201-2014

(15) National Institute for Health and Care Excellence (NICE). Fluocinolone acetonide intravitreal implant for the treatment of chronic diabetic macular oedema after an inadequate response to prior therapy (rapid review of technology appraisal guidance 271). London, England. November 2013. Technology Appraisal Guidance 301.

(16) National Institute for Health and Care Excellence (NICE) technology appraisal guidance. Ranibizumab and pegaptanib for the treatment of age-related macular degeneration. 2012.
